# Changes in central corneal thickness and refractive error after thin-flap laser in situ keratomileusis in Chinese eyes

**DOI:** 10.1186/s12886-015-0083-2

**Published:** 2015-07-29

**Authors:** Ming-Hui Zhao, Qiang Wu, Li-li Jia, Ping Hu

**Affiliations:** Department of Ophthalmology, Shanghai Jiaotong University Affiliated Sixth People’s Hospital, Shanghai,, 200233 China

**Keywords:** Central corneal thickness, Myopia, Non-contact specular microscope, Thin-flap laser in situ keratomileusis, Refractive error

## Abstract

**Background:**

Refractive stability is influenced by alterations in corneal curvature and corneal thickness after laser *in situ* keratomileusis (LASIK). The aim of this study was to analyze the changes of central corneal thickness (CCT) and refractive error following thin-flap LASIK surgery in Chinese eyes.

**Methods:**

One hundred and fifty-eight myopic patients (302 eyes) who underwent thin-flap LASIK surgery were prospectively evaluated. CCT was measured by non-contact specular microscopy before, and 1 day, 1 week, and 1, 3, and 6 months following surgery. Age, refractive error, and optic zone diameter were also recorded.

**Results:**

Preoperatively, the mean CCT was 531.6 ± 24.3 μm. At 1 day, 1 week, and 1, 3, and 6 months after surgery, mean CCTs were 431.4 ± 38.4 μm, 422.6 ± 3 7.8 μm, 427.2 ± 38.0 μm, 434.4 ± 38.2 μm, and 435.6 ± 38.0 μm, respectively. Significant changes were detected in CCT values at each time point after thin-flap LASIK treatment (*P* < 0.05). The mean preoperative spherical equivalent (SE) was −5.73 ± 2.30 diopters (D). At 1 day, 1 week, and 1, 3, and 6 months after surgery, it was 0.26 ± 0.58 D, 0.54 ± 0.52 D, 0.49 ± 0.53 D, 0.45 ± 0.49 D, and 0.37 ± 0.42 D, respectively. The spherical equivalent refraction at 6 months postsurgery was close to the predicted value (0.34 ± 0.30 D). The changes in CCT within 6 months (4.06 ± 9.99 μm) were negatively correlated with age, preoperative refractive error, and optical zone diameter, respectively (r = −0.180, *P* < 0.05; r = −0.187, *P* < 0.001; r = −0.171, *P* < 0.05, respectively). No significant correlation was found between CCT changes and SE changes at different time points, postoperatively.

**Conclusions:**

CCTs decreased significantly at 1 day after surgery, and continued to decline at 1 week after surgery, then increased over time. From postoperative 1 week, SE over time continually shifted to the myopic side.

## Background

Laser *in situ* keratomileusis (LASIK) is a common procedure in refractive surgery [1]. It is known that LASIK can be associated with a rare but visually debilitating refractive instability called ectasia. To prevent corneal ectasia after LASIK, it is recommended that at least 250 μm of residual corneal stromal tissues should be left after ablation. Therefore, the thinner the corneal flap, the thicker the residual corneal stromal bed can be ablated. This is especially important for treating a high degree of myopia. Thin-flap LASIK can create thinner corneal flaps, thicker residual stroma beds, and its safety and efficacy are similar to those of normal LASIK [2].

Refractive stability is influenced by alterations in corneal curvature and corneal thickness, and refractive error usually takes up to 6 months to stabilize after LASIK. The present study aimed to determine the changes in central corneal thickness (CCT) and spherical equivalent (SE) values in Chinese patients following thin-flap LASIK surgery, to evaluate the associations between postoperative CCT changes with age, preoperative SE, and optical zone diameter, and to evaluate the associations between postoperative CCT changes with postoperative refractive error changes.

## Methods

### Subjects

Between May and November 2013, a total of 158 myopic patients (302 eyes) were enrolled in this study. Inclusion criteria were as follows: age ≥ 18 years; stable refraction for at least 2 years; myopia spherical power between −1.50 and −12.00 D; corneal astigmatism less than −4.00 diopters (D); and best-corrected visual acuity (BCVA) ≥ 0.6. Exclusion criteria were as follows: ocular examination suggesting any abnormality in at least one eye (e.g., keratoconus or glaucoma); contact lenses worn within the previous 2 weeks; prior ocular and/or corneal surgery; CCT < 450 μm (measured by ultrasound pachymetry); any active systemic disease that may affect corneal wound healing; or severe dry eye syndrome. Ethical approval for the study was obtained from the Shanghai Clinical Research Center. All subjects were volunteers and informed consents were obtained. The study adhered to the tenets of the Declaration of Helsinki.

### Procedures

Preoperatively, all patients had an ophthalmological examination, including uncorrected visual acuity (UCVA) and BCVA, slit-lamp examination, manifest and cycloplegic refraction measurements (Autorefractor Keratometer Topcon KR-8800; Topcon Ltd., Tokyo, Japan), corneal topography (Allegro Topolyzer™; WaveLight® Inc., Erlangen, Germany), non-contact intraocular pressure (IOP) measurements (Canon TX-F Full Auto Tonometer; Canon Ltd., Tochigiken, Japan), corneal diameter measurements (Allegro Topolyzer™), and wavefront analyses (Allegro Analyzer). CCT in both eyes was measured with a non-contact specular microscope (Topcon SP-2000P; Topcon). In the non-contact specular microscopy study, the subject was positioned with his or her chin in a cup, and the forehead was placed against a headband. CCT and endothelial cell density were measured simultaneously. Only CCT readings were used. Each eye was measured three times. The mean of three readings was obtained for further analyses. All measurements were made by the same ophthalmologist.

LASIK was performed by a single surgeon at a single center using the Moria M2 single-use head 90 μm microkeratome, and subsequent photoablation was conducted with the Allegretto Wave® Eye-Q excimer laser (Wavelight Laser Technologie AG, Erlangen, Germany). All eyes were treated in a routine manner, optical zone diameters ranged from 6.0 mm to 7.0 mm, and the transition zone was 1.5 mm and centered on the patient’s pupil. Postoperatively, patients received prednisolone acetate (1 %) and moxifloxacin (0.5 %) four times daily for 1 week, along with artificial tears as needed.

CCT and refractive errors were measured at 1 day, 1 week, and at 1, 3, and 6 months after surgery. CCT was measured by the non-contact specular microscope. The optical zone diameter was also measured. All measurements were made by the same ophthalmologist (Huijun Liu).

### Data analysis

Statistical analyses were performed using Statistical Package for Social Sciences version 11.0 software (SPSS Inc., Chicago, IL, USA). The one-sample Kolmogorov-Smirnov test was used to test normal distribution. Data were expressed as the mean ± standard deviation (SD). A paired *t*-test was used to compare the preoperative CCT values with 1 day postoperative values, and SE values at 6 months after surgery with the predicted SE values. CCT and SE values at different postoperative time points were compared with each other by using analysis of variance (ANOVA) with Bonferroni *post hoc* tests. The changes in CCT and SE within 6 months (ΔCCT and ΔSE, respectively) were defined as the differences between postoperative 1 day to 6 months for CCT and SE values, respectively (6 months postoperative value minus 1 day postoperative value). Pearson’s partial analyses were used to evaluate the associations between ΔCCT and the following parameters: age, preoperative SE, and optical zone diameter. Pearson’s correlation analyses were performed to examine the relationships between CCT changes and SE changes (different time point postoperative value minus 1 day postoperative value). A *P* < 0.05 was defined as statistically significant.

## Results

All data were distributed normally. The average age of the 158 patients in this study was 29.9 ± 7.5 years (range, 18 to 52 years). 52 patients (102 eyes) were males and 106 (200 eyes) were females. The mean preoperative spherical power was −5.41 ± 0.88 D (range, −1.50 to −12.00 D). The mean cylinder power was −0.76 ± 0.29 D (range, 0.00 to −3.50 D). The mean SE (spherical power + half cylinder power) was −5.73 ± 2.30 D (range, −1.55 to −12.38 D). Baseline preoperative characteristics of patients are shown in Table 1.

**Table 1:** Baseline preoperative characteristics of patients

Parameters	Female	Male	Total
	(*n* = 106)	*(n* = 52)	(*n* = 158)
Age (years)	31.2 ± 7.6	26.2 ± 5.8	29.9 ± 7.5
Spherical power	−5.68 ± 2.21	−4.66 ± 2.06	−5.41 ± 0.88
Cylinder power	−0.73 ± 0.82	−0.73 ± 0.43	−0.76 ± 0.29
SE	−5.77 ± 2.60	−5.06 ± 2.02	−5.73 ± 2.30

Mean value ± standard deviation; SE, spherical equivalent

**Fig. 1:**
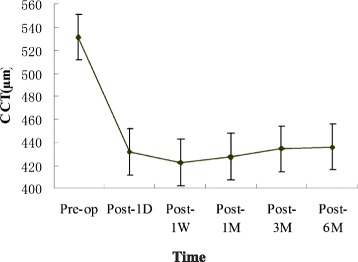
CCT values in pre-surgery, 1 day, 1 week, and 1, 3, and 6 months postsurgery. Central corneal thickness (CCT, in μm) decreased significantly at 1 day (D) after surgery, and continued to decline at 1 week (W). Then it began to increase over time to 6 months (M). Pre-op = preoperative; Post = postoperative. Error bars indicate mean ± SD

No free or incomplete flaps, or flaps with buttonholes occurred in this study. The CCT was found to be 531.6 ± 24.3 μm during the preoperative period. The CCT decreased significantly at 1 day after surgery (431.4 ± 38.4 μm, *P* < 0.05), and continued to decline at 1 week after surgery (422.6 ± 37.8 μm). At 1 month after surgery, CCT values significantly increased (427.2 ± 38.0 μm, *P* < 0.05), and continually increased at 3 and 6 months after surgery (434.4 ± 38.2 μm, *P* < 0.05, and 435.6 ± 38.0 μm, *P* < 0.05, respectively) (Fig. 1). Significant changes were detected in CCT values at each time point after thin-flap LASIK treatment (*P* < 0.05). The mean ΔCCT was 4.06 ± 9.99 μm.

**Fig. 2:**
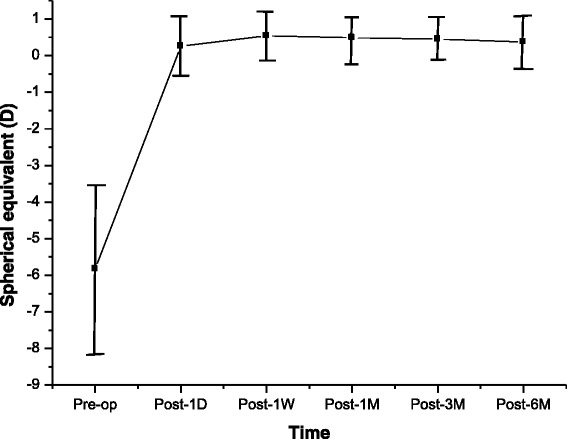
Changes of spherical equivalents (in diopters, D) for up to 6 months after surgery. From 1 week (W) postsurgery, there was a myopic shift over time to 6 months (M). Pre-op = preoperative; Post = postoperative. Error bars indicate mean ± SD

The mean preoperative SE was −5.83 ± 2.30 D. At 1 day and 1 week postsurgery, it was 0.26 ± 0.58 D and 0.54 ± 0.52 D, respectively. There was a myopic shift over time. At 1, 3, and 6 months after treatment, the mean SE was 0.49 ± 0.53 D, 0.45 ± 0.49 D, and 0.37 ± 0.42 D, respectively (Fig. 2). We did not detect any significant change in SE at any time point after LASIK treatment (*P* > 0.05). SE values at 6 months after surgery were similar to the predicted SE values (predicted SE: 0.34 ± 0.30 D, *P* > 0.05). The mean ΔSE was 0.14 ± 0.57 D.

ΔCCT negatively correlated with age, preoperative SE, and optical zone diameter (r = −0.180, *P* < 0.05; r = −0.187, *P* < 0.001; and, r = −0.171, *P* < 0.05, respectively). Table 2 lists increased CCT values of different preoperative SEs. Table 3 lists increased CCT values of different optical diameter zones.

**Table 2:** CCT increased values of different preoperative SE

	1 week	1 month	3 months	6 months
> −3.00 D	−11.28 ± 4.23	−7.70 ± 5.07	0.22 ± 5.64	3.75 ± 7.19
−3.00 D to −6.00 D	−8.41 ± 6.21	−3.86 ± 6.25	1.59 ± 6.13	4.75 ± 7.27
< −6.00 D	−6.94 ± 9.69	−2.11 ± 6.15	4.44 ± 7.43	6.51 ± 9.61

Mean value ± standard deviation (μm); D = diopters; CCT = central corneal thickness; SE = spherical equivalent. Values are different postoperative time point CCT values minus CCT values at 1 day postsurgery

**Table 3:** CCT increased values at different optical diameter zones (μm)

zone	1 week	1 month	3 months	6 months
6.0 mm	−6.76 ± 6.03	−2.83 ± 6.14	5.16 ± 7.46	4.84 ± 7.51
6.5 mm	−7.04 ± 6.34	−3.61 ± 6.37	4.57 ± 8.10	5.28 ± 11.58
7.0 mm	−8.77 ± 6.68	−5.64 ± 6.68	1.28 ± 6.40	1.38 ± 7.81

Values are different postoperative time point CCT values minus CCT values at 1 day postsurgery at the three indicated zones. CCT = central corneal thickness

We did not detect any significant correlations between CCT changes and SE changes (different time point postoperative value minus 1 day postoperative value), when measured at different examination points (r = −0.087, *P* = 0.054). No regression occurred within 6 months. All patient UCVAs after surgery were better than 16/20.

## Discussion

Many types of methods can be used to measure corneal thicknesses, such as ultrasound pachymetry, anterior-segment optical coherence tomography (AS-OCT), Pentacam measurements, Orbscan measurements, and non-contact specular microscopy techniques. However, the values are not always consistent between the different instruments. In a long-term follow-up study, it was recommended to uniformly use the same kind of measuring instrument [3–5]. Non-contact specular microscopy is a commonly used method for corneal thickness measurement, with advantages including noninvasiveness, ease of operator use, and good examiner-independent reproducibility [6]. Non-contact specular microscopy can be an ideal technique for studying the corneal thickness changes after photorefractive keratectomy, because it is noninvasive, which is especially important during the early postsurgery stage. Changes of CCT values are very important for observation of corneal tissue healing responses after surgery.

In this study, we used non-contact specular microscopy to follow-up the CCT changes within 6 months post thin-flap LASIK treatment. We found that CCT values declined significantly at 1 day postsurgery, and continued to decline at 1 week. CCT values began to increase over time. Several processes occurred during the early postoperative period, including resorption of fluid introduced by intraoperative irrigation, biomechanical hydration shift, epithelial thickness modulation in response to laser ablation, and interface reflectivity changes. However, in many reported cases, the systematic changes are small after 1 week [7], and the posterior stroma is significantly thickened after 1 week postsurgery [8, 9]. Peng et al. [10] reported that just after LASIK surgery, keratocytes were activated by cytokines that induced collagen fiber synthesis. Keratocyte activation was strongest at 1 to 2 weeks, and persisted until 3 months after LASIK surgery [8, 9]. This could cause the increase in posterior stromal thickness, and may be why the CCT values continued to increase after 1 week postsurgery.

In this study, we show that from 1 week postoperatively, SE continually shifted to the myopic side over time. Corneal wound repair is believed to be a contributing factor in the gradual increase of corneal thickness and postoperatively in the development of refractive regression [11, 12]. Epithelial hyperplasia after photorefractive keratectomy (PRK) has been suggested to contribute to the loss of the postoperative refractive effect [13–16]. However, after LASIK surgery, epithelial changes were significantly reduced, and were not the main cause for refractive instability [17]. Avunduk et al. reported no significant changes in epithelial thicknesses at any time point after LASIK treatment [18]. MØller-Pedersen et al. demonstrated activated keratocyte-mediated rethickening of the photoablated stroma of myopic individuals [11]. They further demonstrated that corneal rethickening caused myopic regression mediated almost solely by stromal rethickening; only a minor contribution appeared to originate from restoration of the postoperative epithelial thickness [11]. In the present study, we found a significant increase in the CCT between 1 week and 6 months after surgery (422.6 μm versus 435.6 μm). The spheroequivalent refraction changed to the myopic side between these time points (0.54 D versus 0.37 D), but the difference did not reach statistical significance, and no significant correlation was detected between the SE value changes and the CCT value changes at different examination time points. Normally, it would be expected that a 10 μm to 15 μm rethickening of the posterior stroma would produce a 1 D myopic shift. However, in the present study, the much greater rethickening created only a small amount of refractive change. It is difficult to explain this refractive change with activated keratocyte-mediated rethickening of the photoablated posterior stroma. Avunduk et al. suggested that the most probable explanation is that the refractive change is induced by different refractive characteristics of activated keratocytes during LASIK surgery. Therefore, the anterior and posterior curvature and the refractive index may be shifting postsurgery, and these factors may also play a role [18]. However, we do not have any corneal curvature measurements to support this hypothesis.

Li et al. found that CCT increased within 12 months after LASIK surgery, and was correlated with age, preoperative SE, and corneal bed thickness (r = −0.554, r = 0.382, r = −0.352, respectively) [19]. Our present observations were similar, however, in our study the correlations were all weak (r < 0.2). The reason for this difference is unclear, and further study is needed to confirm this finding. In elderly patients, tissue regeneration ability is reduced therefore the possibility of refractive regression is lower. It is not recommended to overcorrect on LASIK in patients over 40 years old [14]. To prevent the occurrence of refractive regression after surgery in patients with high myopia, it is recommended to expand the optical zone diameter as large as possible, leaving sufficient corneal stromal tissues. We also retained a certain degree diopter that was corrected after surgery with eye glasses [15].

## Conclusion

In conclusion, CCT decreased significantly at 1 day after surgery, and continued to decline at 1 week after surgery, then it increased over time. From 1 week postoperative, SE continually shifted to the myopic side, with increased time postsurgery.

## Abbreviations

LASIK: Laser *in situ* keratomileusis
CCT: Central corneal thickness
SE: Spherical equivalent
UCVA: Uncorrected visual acuity
BCVA: Best-corrected visual acuity
SD: Standard deviation
ANOVA: Analysis of variance
AS-OCT: Anterior-segment optical coherence tomography
